# Assessment of antibody responses against SARS-CoV-2 in unvaccinated individuals and vaccinees from Omicron-BA.2 infection in Zhaoqing, Guangdong Province, China

**DOI:** 10.1186/s12985-022-01912-0

**Published:** 2022-11-25

**Authors:** Huan Zhang, Yushan Jiang, Hanqing Tan, Lirong Zou, Zhonghua Zheng, Yushi Huang, Shujian Lin, Lebin Su, Jianxiang Yu, Xiaoling Deng, Jianfeng He, Chang Liu, Chenguang Shen, Baisheng Li

**Affiliations:** 1 Guangdong Workstation for Emerging Infectious Disease Control and Prevention, Chinese Academy of Medical Sciences, Guangdong Provincial Center for Disease Control and Prevention, Guangzhou, Guangdong China; 2 Biosafety Level-3 Laboratory, School of Public Health, Southern Medical University, 510515 Guangzhou, China; 3 Microbiological Laboratory, Zhaoqing Center for Disease Control and Prevention, Zhaoqing, China

**Keywords:** Omicron-BA.2, Breakthrough infection, Antibody responses, IgM, Neutralization antibody

## Abstract

Currently, the majority of the global population has been vaccinated with the COVID-19 vaccine, and characterization studies of antibodies in vivo from Omicron breakthrough infection and naive infection populations are urgently needed to provide pivotal clues about accurate diagnosis, treatment, and next-generation vaccine design against SARS-CoV-2 infection. We showed that after infection with Omicron-BA.2, the antibody levels of specific IgM against the Wuhan strain and specific IgG against Omicron were not significantly elevated within 27 days of onset. Interestingly, in this study, the levels of humoral immunity against Omicron-specific IgM were significantly increased after breakthrough infection, suggesting that the detection of Omicron-specific IgM antibodies can be used as a test criterion of Omicron breakthrough infection. In addition, we observed that serums from unvaccinated individuals and the majority of vaccinated infections possessed only low or no neutralizing activity against Omicron at the onset of Omicron breakthrough infections, and at the later stage of Omicron-BA.2 breakthrough infection, levels of neutralization antibody against the Wuhan and Omicron strains were elevated in infected individuals. The findings of this study provide important clues for the diagnosis of Omicron breakthrough infections, antibody characterization studies and vaccine design against COVID-19.

## Introduction

The emergence of severe acute respiratory syndrome coronavirus 2 (SARS-CoV-2) disease (COVID-19) was first discovered in Wuhan in China at the end of 2019 and was announced as a global pandemic since the beginning of 2020 by the World Health Organization (WHO). The virus has undergone multiple mutations during transmission, which has led to the emergence of several important variants of concern (VOCs), such as Alpha, Beta, Gamma, Delta, Omicron-BA.1, and Omicron-BA.2 [1]. Omicron various versions, like BA.2, BA.4, and BA.5, are presently the most widespread strains globally [2]. In comparison to the original SARS-CoV-2 Wuhan strain and variants such as Alpha and Delta, the Omicron variant contains more amino acid mutations in viral antigenicity, raising concern over the virus escaping from immune protection induced by COVID-19 vaccines, antibodies in patients recovering from early COVID-19 infection, and the majority of marketed monoclonal antibody drugs, so Omicron breakthrough infection has been occurring on a regular basis around the world [3–5]. Studies on the characteristics of humoral immune response in individuals infected by SARS-CoV-2 have been an important research topic in the past two years. Currently, the majority of the global population has been vaccinated with the COVID-19 vaccine (SARS-CoV-2 Wuhan antigen) or infected by the original SARS-CoV-2. Characterization studies of antibodies in vivo from Omicron breakthrough infection (vaccinated or SARS-CoV-2 infected) and naive infection (first-time infected with SARS-CoV-2) populations are urgently needed to provide pivotal clues about accurate diagnosis, treatment, and next-generation vaccine design against SARS-CoV-2 infection. In April 2022, an outbreak of the Omicron-BA.2 occurred in Zhaoqing, Guangdong Province, China, and in this cohort study, we assessed the antibody profile of serum samples from infected individuals in this outbreak.

## Method

### Study design

In this study, serum samples were taken randomly from 32 infected patients with 159 serum samples at various stages of Omicron-BA.2 infections discovered in Zhaoqing City, Guangdong Province, China, from March 25, 2022, to April 2, 2022, collected range from 1 to 27 days after the onset of infection. This study followed the Strengthening the Reporting of Observational Studies in Epidemiology (STROBE) reporting guideline. All the individuals had no history of earlier COVID-19 infection.Vaccinated individuals who had 2 doses of vaccination had infection with BA.2 after 4 to 9 months, while vaccinated individuals who had 3 doses of vaccination had infection with BA.2 after 2 to 5 months in this study.

### Enzyme-linked immunosorbent assay (ELISA)

Microtiter plates (Sangon Biotech) were coated with 1 µg/mL of each recombinant SARS-CoV-2 RBD of the spike protein (100 µL per well) overnight at 4 °C. The plates were then washed twice with phosphate buffer saline (PBS) containing 0.1% v/v Tween-20 (PBST) and blocked with blocking solution (PBS containing 5% w/v bovine serum albumin) overnight at 4 °C. The plates were then washed once with PBST. The sera were diluted 10,000-fold by PBS, and serial 2-fold dilutions of sera were added to the wells and incubated at 37 °C for 60 min. After five washes, 100 µL of horseradish peroxidase (HRP)-conjugated goat anti-human IgG or IgM antibody solution (Sangon Biotech) was added to each plate, respectively, and incubated at 37 °C for 60 min. After five washes, 100 µL of tetramethylbenzidine (TMB) substrate (Sangon Biotech) was added at room temperature in the dark. After 15 min, the reaction was stopped with a 2 M H_2_SO_4_ solution. The absorbance was measured at 450 nm. All samples were run in triplicate. The ELISA titers were determined by endpoint dilution.

### Serum-neutralization assay

10^4^ Vero cells were seeded 24 h before the infection in a 96-well plate (Costar). On the day of infection, the cells were washed twice with a cell culture medium. Sera from individuals were incubated at 56℃ for 30 min, and then two-fold (several times). Aliquots (40 µL) of diluted sera (from 20-fold to 5120-fold dilutions) were added to 50 µL of cell culture medium containing 100 tissue culture infective dose (TCID_50_) of wild type or Omicron-BA.2 virus strain (isolated from Guangdong Provincial Center for Disease Control and Prevention) on a 96-well plate and incubated at 37℃ for 2 h in CO_2_ 5% v ⁄ v. Virus serum mix was then added to cells in 96-well plates and plates were incubated at 37℃ with microscopic examination for cytopathic effect (CPE) after the 5-day incubation. The highest dilution of serum that showed inhibition activity of SARS-CoV-2 was estimated as the NT titer. NT assays were performed in triplicate with negative control sera. The threshold of neutralization activity is NT ≥ 4.

### Statistical analysis

With the Kruskal-Wallis test, multiple comparisons were conducted. The two-tailed Wilcoxon signed-rank matched-pairs test was used to compare paired comparisons (α = 0.05). In 2-tailed testing, P < 0.5 was deemed statistically significant. Multiple comparisons were performed using the Welch’s t-test. Two-tailed Wilcoxon signed-rank matched-pairs test was used to compare paired comparisons. P < 0.5 was considered to be statistically significant in 2-tailed tests. All statistical analysis was conducted using Prism version 9.3.0 (Graphpad).

## Results

**Fig. 1:**
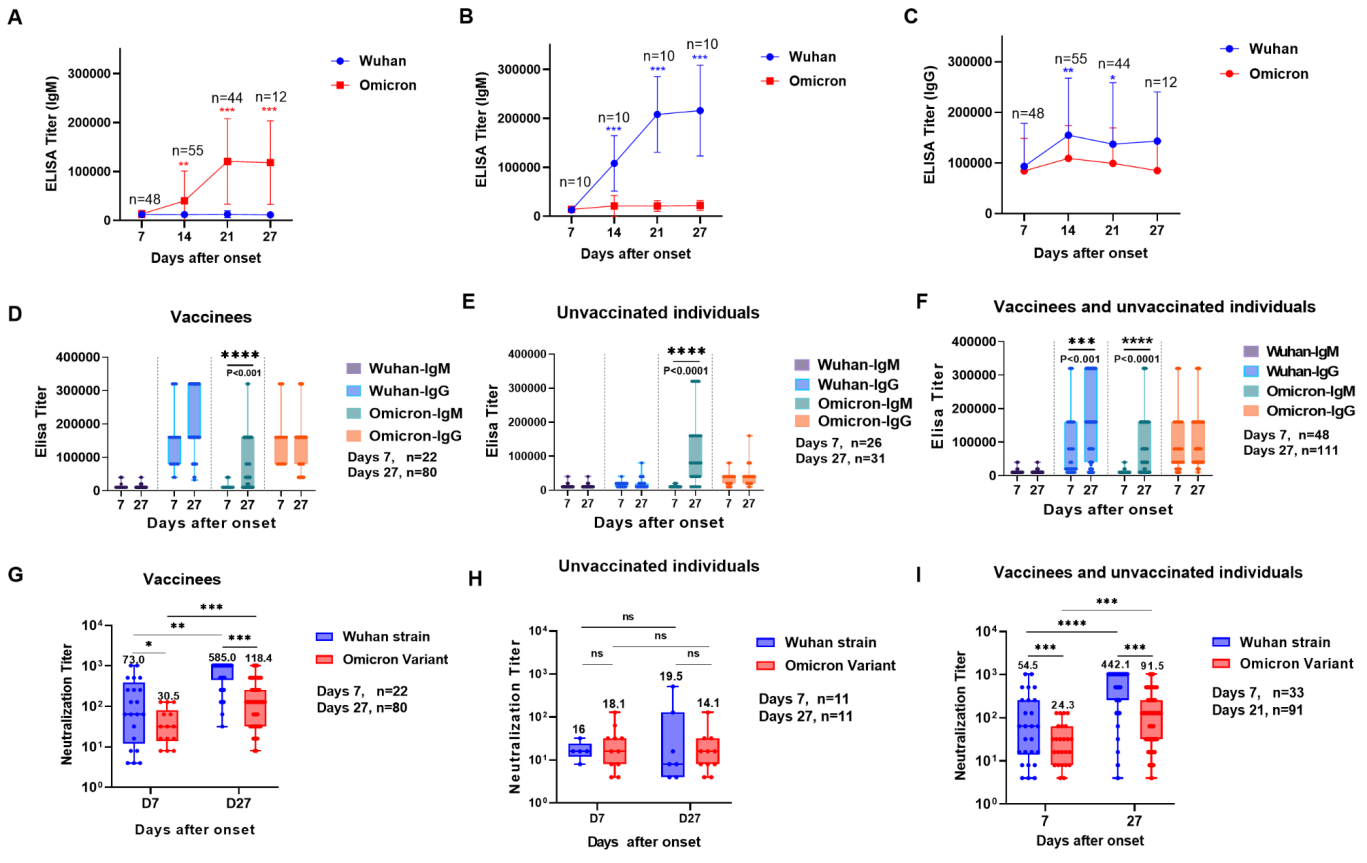
Assessment of antibody responses against SARS-CoV-2 in unvaccinated individuals and vaccinees from Omicron-BA.2 infection (A-B) Kinetics of anti-RBD IgM antibody titers of sera from Omicron-infected individuals (A) and Wuhan strain-infected individuals (B) against SARS-CoV-2 Wuhan strain and Omicron-BA.2 strain during 7–27 days after onset using the ELISA assay. (C) Kinetics of anti-RBD IgG antibody titers of sera from Omicron-infected individuals against SARS-CoV-2 Wuhan strain and Omicron-BA.2 strain during 7–27 days after onset using the ELISA assay.The titers were calculated at 450nm (OD50), and serum samples from 7 days, 8 to 14 days, 15 to 21 days, and 22 to 27 days after onset were tested respectively. (D-F) Dynamic change of anti-RBD IgM and IgG antibody titers against SARS-CoV-2 Wuhan strain and Omicron-BA.2 strain in vaccinees of inactivated COVID-19 vaccine (panel D) or unvaccinated individuals (panel E) or all COVID − 19 patients (panel F) in the 0–7 days or 8–27 days after onset. (G-I) Dynamic change of neutralization antibody titers against authentic SARS-CoV-2 Wuhan strain and Omicron-BA.2 variant in vaccinees of inactivated COVID-19 vaccine (panel G) or unvaccinated individuals (panel H) or all COVID − 19 patients (panel I) in the 0–7 days or 8–27 days after onset. Geometric mean titers (GMT) of neutralizing antibodies were shown. The sample size for each panel was shown as n. P values were obtained from a comparison between the two treatment groups using t-tests for long-transformed antibodies or two-sided χ2 tests for categorical data. Multiple comparisons of antibody titers were performed using the Welch’ t-test

A total of 32 Omicron-infected patients, ranging from 14 to 63 years old, with a mean age of 36 years old, were identified, 12 of whom were male. Nineteen infected patients received inactivated vaccine (BBIBP-CorV or CoronaVac), of which 13 received three doses of inactivated vaccine and six received two doses of inactivated vaccine (Table 1). Among these 32 infected patients, both IgM and IgG antibodies specific to the RBD protein of Wuhan and Omicron strains were at undetectable levels within 7 days of symptom onset, the titers of Omicron-specific IgM antibodies were significantly elevated after day 7 of the onset, peaking at 14–21 days after onset. However, no significant increase in the titers of Wuhan-specific IgM levels was observed within 27 days of onset (Fig.1A). On the other hand, 40 sera from individuals infected with Wuhan virus strain were used as controls, it was found that Wuhan strain-specific IgM antibodies were present in sera within 27 days after onset, in contrast, within 27 days after onset, Omicron-specific IgM has not been obviously detected (Fig.1B). Since most of the patients had been vaccinated against COVID-19, most of them had detectable IgG titers against Wuhan and Omicron strains within 7 days of symptom onset, the specific IgG titer against Wuhan strain increased significantly within 8–14 days of symptom onset and peaked within 14 days, in contrast, the specific IgG titer against Omicron strain did not elevate significantly during the observation (Fig.1C). Specifically, for individuals who have been vaccinated against COVID-19, the titers of IgM against Wuhan strain, IgG against Wuhan strain, and Omicron-specific IgG have no significant difference between the early and later stages of symptom onset (Fig.1D). Similarly, for unvaccinated individuals, only Omicron-specific IgM titers were significantly elevated with time delay in the time range of the assay (Fig. 1E). For overall patients with COVID-19, Wuhan strain-specific IgG and Omicron-specific IgM antibody titers are significantly increased at the later stage of the disease, while other antibody indicators do not change significantly during different stages (Fig.1F). In addition, we tested the titers of neutralizing antibodies against Wuhan and Omicron strains in COVID-19 patients at different stages after onset. In the early stages of onset (0 day − 7th day after onset), neutralizing antibodies against the Wuhan strain were detected in each vaccinated individual (geometric mean titer [GMT] 73.0 [4.0-1024.0]), and the GMT was 30.5 (4.0-1024.0) against Omicron-BA.2 strain. In the late stages of infection (8th day-27th day after onset), serums from Omicron breakthrough infected individuals had significantly higher antibodies against the Wuhan and Omicron strains when compared to those in the early stages, with a mean of GMT 585.0 (32.0-5142.0) against Wuhan strain and GMT 118.4 against Omicron strain (8.0-512.0). The titer of specific neutralizing antibodies against Wuhan strain was significantly higher than that of specific neutralizing antibody against Omicron at both early and late stages after onset (Fig.1G). While among the 13 unvaccinated individuals, only the sera from three infected individuals (3/13) showed neutralization activity against the Wuhan strain with a GMT 16 (8.0–16.0), and sera from four infected individuals (4/13) revealed neutralization activity against the Omicron strain with GMT 18.1 (4.0-128.0) (Fig.1H). In the later stage after onset (8th day-27th day of infection), neutralizing antibody titers against Wuhan and Omicron strains did not increase significantly when compared to those in the early stage, sera from three infected individuals (3/13) had neutralizing potency against the Wuhan strain (GMT 19.5), and all unvaccinated infected individuals produced neutralizing antibodies against Omicron at the late stage (GMT 14.1) (Fig.1I).

**Table 1:** Demographic characteristics in individuals from Omicron variant infections

Characteristic		Number	(%)
Reported sex	Female	20	62.5%
	Male	12	37.5%
Age	14–63	32	NA
COVID-19 vaccination	Vaccinees	19	59.4%
	Unvaccinated individuals	13	40.6%
	Total	32	NA

The table includes all individuals infected by BA.2 (n = 32) for which the serum samples from them were collected from 1 to 27 days after symptom onset

## Discussion

For individuals who have been vaccinated with COVID-19 or have been infected with SARS-CoV-2, serum from them already contains specific IgG and IgM antibodies explicitly targeting the Wuhan strain of SARS-CoV-2. The results of this study showed that after breakthrough infection with Omicron-BA.2, the antibody levels of specific IgM against the Wuhan strain and specific IgG against Omicron were not significantly elevated within 27 days of onset. While for the unvaccinated population, the aforementioned antibody levels also did not show enhancements during the observational period, indicating that the antibodies cannot be used as indicators for Omicron breakthrough infection. Specific IgM antibodies to the Wuhan strain were not significantly increased, which may be due to the fact that the currently popular Omicron variant processes many mutations on the key antigenic spike protein, Omicron-specific IgM antibodies possessed weak reaction activity to the Wuhan strain. Therefore, current vaccines based on the original Wuhan strain confer very little protection against Omicron variant [6]. Interestingly, in this study, the levels of humoral immunity against Omicron-specific IgM were significantly increased after breakthrough infection, suggesting that the detection of Omicron-specific IgM antibodies can be used as a test criterion of Omicron breakthrough infection. As we know, IgM antibodies are produced at a very early stage of infection, then the antibody isotype switch from IgM to IgG usually occurs with the progression of the humoral immune response development [7]. Therefore, IgM antibodies are generated in the early stages of infection, then IgG is a signal for the latter stages of infection. This could be the reason for the lack of significantly increased affinity to IgG antibodies against BA.2 in our research. As the observation period was relatively short, if observation times were extended, the IgG will be increased later. In addition, we observed that serums from unvaccinated individuals and the majority of vaccinated infections (23/32) possessed only low or no neutralizing activity against Omicron (GMT 24.3) at the onset of Omicron breakthrough infections, suggesting that Omicron-BA.2 can largely escape vaccine-induced immune protection, which produces breakthrough infections, and these findings are consistent with other publications [8–11]. At the later stage of Omicron breakthrough infection, antibody neutralization levels against the Wuhan strain were elevated in infected individuals. Similarly, the mean neutralization titers of late serum against Omicron were significantly higher than those of early Omicron infection or pre-infection serum, suggesting that breakthrough infection with the Omicron strain enhances the specific antibody response to SARS-CoV-2 titers, and the induced neutralizing antibodies may be able to neutralize Omicron and Wuhan strains broadly. These results suggest that Omicron-associated antigens may have potential as candidate antigens for vaccine booster shots, which is consistent with our previous findings on Omicron booster shots at the animal level [12]. Besides that, when comparing the late stage of infection to the early stage of infection in the immunized group, no significant increases in binding antibody titers against the Wuhan strain were seen, but there was a substantial rise in neutralizing antibodies. This indicates that breakthrough Omicron infection could not increase the total antibody titers against the Wuhan strain, but it might improve the neutralizing antibody titers during our observation. This inspires us when evaluating immune responses to COVID-19 booster vaccinations not only based on the binding antibody titers but also by neutralizing antibody levels. In conclusion, the findings of this study provide important clues for the diagnosis of Omicron breakthrough infections, antibody characterization studies, and the design of next-generation vaccines for COVID-19. This study has a limitation, due to the insufficient number of samples in this study, we could not describe the difference in antibody response in vaccinees who had 3 doses and 2 doses with breakthrough infections, further elaborating on the comparative analysis of the antibody responses between these different vaccinees would be helpful.

## Data Availability

The data generated and/or analyzed during the current study are available from the corresponding authors on reasonable request.
